# Target Recognition in Infrared Circumferential Scanning System via Deep Convolutional Neural Networks

**DOI:** 10.3390/s20071922

**Published:** 2020-03-30

**Authors:** Gao Chen, Weihua Wang

**Affiliations:** National Key Laboratory of Science and Technology on ATR, National University of Defense Technology, Changsha 410073, China; atrwwh@126.com

**Keywords:** infrared circumferential scanning system, target recognition, deep convolutional neural networks, data augmentation, transfer learning, bounding box regression, loss function

## Abstract

With an infrared circumferential scanning system (IRCSS), we can realize long-time surveillance over a large field of view. Recognizing targets in the field of view automatically is a crucial component of improving environmental awareness under the trend of informatization, especially in the defense system. Target recognition consists of two subtasks: detection and identification, corresponding to the position and category of the target, respectively. In this study, we propose a deep convolutional neural network (DCNN)-based method to realize the end-to-end target recognition in the IRCSS. Existing DCNN-based methods require a large annotated dataset for training, while public infrared datasets are mostly used for target tracking. Therefore, we build an infrared target recognition dataset to both overcome the shortage of data and enhance the adaptability of the algorithm in various scenes. We then use data augmentation and exploit the optimal cross-domain transfer learning strategy for network training. In this process, we design the smoother L1 as the loss function in bounding box regression for better localization performance. In the experiments, the proposed method achieved 82.7 mAP, accomplishing the end-to-end infrared target recognition with high effectiveness on accuracy.

## 1. Introduction

Objects with a temperature above absolute zero can continuously emit electromagnetic radiation into outer space. At room temperature, these radiations mainly concentrate in the infrared band. The infrared radiation emitted or reflected by the target is captured by thermal imagers to obtain high-contrast imaging results, which are usually grayscale images. Compared with visible cameras, the thermal infrared imager can work at night and has a specific ability to distinguish the true and false targets, because it relies on the difference in temperature and emissivity between the target and the ground. Compared with radar images, infrared images are more recognizable to human eyes because of the shorter wavelength of infrared radiation. Additionally, the thermal imager has passive characteristics, only receiving the target radiation, and does not need to transmit a signal, which means high concealment. With the advantages of all-day work, high concealment, and sensitivity, thermal infrared imagers are widely used to collect information in a variety of complex environments, and to achieve 24-h surveillance, especially in the defense system, which can provide supports for battlefield decision-making in modern warfare [1,2].

The infrared circumferential scanning system (IRCSS), equipped with a long linear infrared focal plane array (IRFPA), performs circum-sweep motion under precise servo control to realize circum- sweep imaging at multiple pitching angles [3]. Moreover, the horizontal coverage is close to 360 degrees, greatly expanding the detection range of infrared detectors. The typical imaging resolution of IRCSS can reach 768×120,000, which is much higher than that of the conventional forward-looking infrared system [4]. With the rapid development of image processing technology, the IRCSS has the capabilities of searching over a large field of view, long-range target automatic recognition, multi-target high-precision tracking, and integration with mechanical control systems.

When the field of view expands, there will be more interference in the imaging results of the IRCSS, such as clouds in the low-altitude background, and mountains and trees in the ground background, all of which make target recognition more challenging. However, most of the previous works only finished the target detection [5,6]. Our method is to upgrade the function of the IRCSS. Target recognition involves two levels of understanding of the target. Firstly, where is the target? We need to perform detection, deciding whether the target is in range, and localize it if it is. Secondly, what is the target? We need to perform identification, classifying the target. In the past, traditional hand-crafted methods took different algorithms for two subtasks—for example, threshold segmentation [7] for target localization, a histogram of oriented gradients (HOG) [8] for features extraction, and a support vector machine (SVM) [9] for classification. The representative work in this paradigm is the deformable part models (DPM) [10]. However, independence between algorithms becomes an obstacle to further enhance recognition performance. Since AlexNet [11] became the preferred option in ILSVRC 2012, there has been a strong interest in deep learning for computer vision, especially the deep convolutional neural network (DCNN). The DCNN has the structure of stacking various blocks, such as convolution layers, activation functions, pooling layers, and fully connected layers, which endows the network with a powerful feature extraction ability to automatically and adaptively obtain deep semantic information. In the target recognition field, DCNN-based methods utilize a network to accomplish two subtasks, realizing end-to-end processing. In learning-based models, DCNN-based methods need large-scale datasets, including images and annotations, to train the networks with a strong generalization ability. Owing to the access to large-scale datasets for the public, such as ImageNet [12], COCO [13], VOC [14], the performance of DCNN-based methods on visible images has been dramatically improved. However, in the infrared field, publicly available datasets are mostly used for target tracking, which are usually sequences of infrared images. Unfortunately, these datasets are unsuitable for infrared target recognition. 

**Figure 1:**
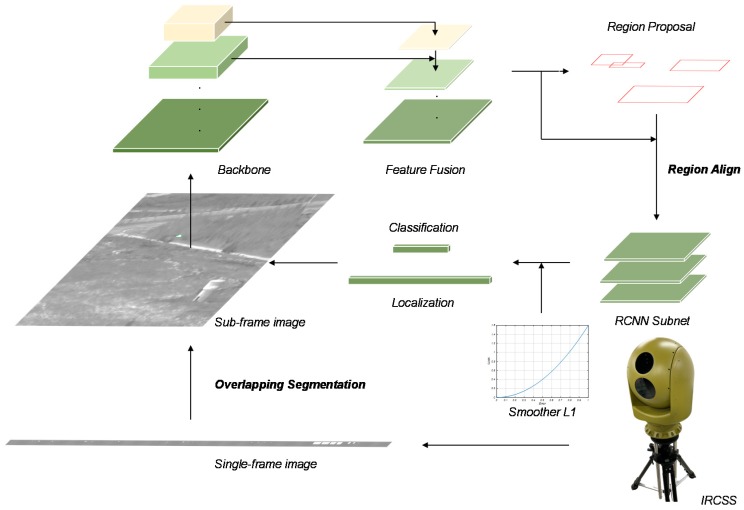
The overall architecture of the proposed method to address target recognition in the infrared circumferential scanning system (IRCSS). We perform the overlapping segmentation on the single-frame image of IRCSS. After getting the sub-frame image, it is sent to the recognition network. The backbone structure is detailed in Section 4. The feature fusion follows the design in the feature pyramid network (FPN) [15]. The region proposal network (RPN) and the region convolutional neural network (RCNN) subnet follows the design of the Faster RCNN [16]. The region proposals are processed by RoI align [17]. In the bounding box regression for target localization, the loss function is the smoother L1.

In this paper, as shown in Figure 1, we propose a DCNN-based method to address target recognition in the IRCSS. In the experiments, results demonstrated the effectiveness of the proposed method on accuracy with 82.7 mAP. The contributions in this paper can be summarized as follows:

1. We realize end-to-end target recognition on high-resolution imaging results of the IRCSS via the DCNN.
2. We build an infrared target recognition dataset to both overcome the shortage of data and enhance the adaptability of the algorithm in various scenes, including two types of targets in seven types of scenes with two types of aspect orientations, four types of sizes and twelve types of contrasts.
3. We design a loss function called the smoother L1 in the bounding box regression for better localization performance.

The rest of the paper is organized as follows. Section 2 reviews related works about target recognition and tracking algorithms. In Section 3, we describe the methodology of this paper. Section 4 is the experiment part, including experiment details, results, and analysis. Section 5 provides the conclusion and a plan for future work.

## 2. Related Work

### 2.1. Target Recognition and Tracking in Infrared Images

Thermal infrared sensors are not influenced by illumination variations and shadows, and objects can be distinguished from the background as the background is normally colder [18]. Considering these advantages and the demand for realistic, computer vision tasks in infrared images have emerged, such as target recognition and tracking.

Target recognition can be divided into two subproblems: target detection and identification—in other words, localizing the target in the image and figuring out its category. Target identification can be further divided into feature extraction and classifier design. The sliding window approach was the most straightforward way to localized the target [19]. It slid a window over the image to obtain image patches, and the target recognition model was then used to classify each patch covered by the window. To overcome the limitations of expensive computation of the sliding window approach, the selective search [20] approach was used to segment the image into original regions using the algorithm in [21] and then grouped similar regions based on color, texture, size, and shape compatibility. This process was repeated until the number of iterations was reached. For target identification, several methods have been proposed. In [22], a sparse representation-based classification (SRC) algorithm was proposed for infrared target recognition. In [23], the HOG and bag-of-words (BoW) was applied to further improve performance. With respect to the IRCSS, previous methods have finished the detection of targets. A time-domain multi-frame cumulative difference algorithm was proposed to detect the dim and small target in the large field of view and complicated background [6]. In [5], a rough-to-meticulous target detection algorithm was proposed for panorama infrared images. In the rough detection phase, the integrating processing of morphological filtering and interframe differences was utilized to pick up suspected targets most rapidly from high-resolution images and suspected target image slices were generated. In the meticulous detection phase, permanent false alarm adaptive threshold method and feature fusion were adopted to eliminate false alarm and generate a trajectory for the real targets.

Based on the annotation of a target only on the first frame of the video, target tracking aims to estimate a moving trajectory [24]. A discriminative correlation filter (DCF)-based tracker learns a correlation filter from annotations to discriminate the target from the background [25]. Even after several years, this branch is still flourishing in the tracking field. With respect to infrared tracking, in VOT-TIR2017 [26], which is a challenge on tracking in thermal infrared sequences, the winner DSLT [27] applied optical flow and extended Struck [28] with the ability to learn from dense samples and high dimensional features. The top accuracy tracker SRDCFir [29] introduced a spatial regularization function that penalized filter coefficients residing outside the target region to alleviate the periodic assumption when using circular correlation. Another branch of infrared tracking fuses infrared images and visible images. In the corresponding VOT-RGBT2019 challenge [30], the DCF-based tracker JMMAC [30] designed a robust RGBT (RGB and thermal) tracker that combined motion cues and appearance cues. The motion cue was inferred from key-point-based camera motion estimation and a Kalman filter applied to object motion. The appearance cues are generated by an extension of the efficient convolution operators (ECO) model [31]. In this paper, we are interested in target recognition. In the next part, we introduce an overview of DCNN-based target recognition methods.

### 2.2. DCNN-Based Target Recognition

Feature extraction plays an important role in both target recognition and tracking. The traditional hand-crafted features have been used in various modalities images [32]. Over the past few years, DCNN-based method has outperformed the traditional approaches in various computer vision domains, such as image classification, target recognition, and semantic segmentation, because of the strong ability of feature extraction.

DCNN-based methods utilize a single network to accomplish two subtasks in target recognition. According to the number of stages, they can be divided into two branches: two-stage methods and one-stage methods. 

The two-stage methods, which are also known as classification-based methods, divide the recognition into two stages. In the first stage, the network selects target region proposals from the predefined boxes over the image and refines their position coordinates. This process can be regarded as binary classification. Each anchor is classified as the target-in or target-out. In the second stage, each proposal is classified and refined again. In general, benefiting from a region proposal network and two-time position refinement, two-stage methods have relatively higher precision. Faster RCNN [16] firstly finalized the above-mentioned workflow. After that, the feature pyramid network (FPN) [15] was proposed to fuse semantic information from deep layers and location information from shallow layers. Cascade RCNN [33] utilized three thresholds for better region proposals in the first stage. DetNAS [34] adopted the neural architecture search [35] to find the optimal architecture of the recognition network. CBNet [36] proposed a strategy of compositing connections between the adjacent backbones to build a more powerful backbone network than ResNet [37] and ResNeXt [38], which achieved the best 53.3 mAP on the COCO benchmark [13].

Meanwhile, the one-stage method can directly predict coordinates and categories of the targets by a multi-tasks loss function, which is also called the regression-based method. The basic architecture consists of the backbone network and detection subnet. Owing to less computation, one-stage methods are mostly proposed to achieve a faster speed of recognition. If the limit is 20FPS, YOLO [39] is the first to realize real-time target recognition. As an improved version, YOLO v3 [40] significantly improved the precision while maintaining the speed and has been widely used in realistic circumstances. SSD [41] was used to make predictions on feature maps of different scales. EfficientDet [42] utilized EfficientNet [43] and BiFPN to develop a family of networks, among which EfficientDet-D7 achieved 51.0 mAP on the COCO benchmark [13].

Most DCNN-based target recognition algorithms are mostly proposed for RGB images. When used in the infrared system, diversities between characteristics of images may cause more problems, making recognition more challenging. In [44], a DCNN-based detector was designed in the infrared small unmanned aerial vehicle (SUAV) surveillance system by the laterally connected multi-scale feature fusion approach and densely paved predefined boxes. In [45], SVM and DCNN classification for infrared target recognition were compared. Some literature only takes CNN as feature extractors. In [46], CNN cooperated with the difference of Gaussian (DoG) to recognize the target. In [47], a compact and fully CNN was trained with synthetic data because of the shortage of infrared data. The trained network was used to address target recognition in an infrared defense system. In this paper, we propose a two-stage method to accomplish end-to-end target recognition in the IRCSS.

## 3. Methodology

We propose a DCNN-based two-stage method for target recognition in the IRCSS. Figure 1 shows the overall architecture. To be specific, owing to the large size of the single-frame image in the IRCSS, we perform overlapping segmentation on it to obtain sub-frame images. We then build an infrared target recognition dataset to both overcome the shortage of data and enhance the adaptability of the algorithm in various scenes. Furthermore, we adopt data augmentation to expend the dataset and exploit the optimal cross-domain transfer learning strategy for the train. In the network, we design a novel loss function in bounding box regression for target localization, called the smoother L1.

### 3.1. Sub-Frame Images of the IRCSS

An IRCSS consists of two parts: an infrared detector with a long linear IRFPA and a mechanical structure. Under precise control by a servomotor, the detector performs uniform rotation to obtain circumferential images. Compared with the simple staring thermal imager, the system can provide a large field of view and continuous circumferential images, which can be applied in environmental monitoring and night navigation.

**Figure 2:**
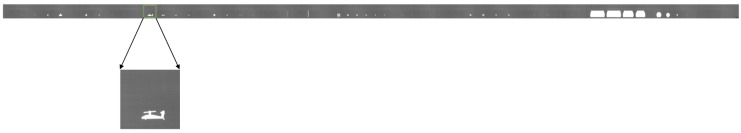
The single-frame image obtained by the IRCSS. In this paper, its size is 768×40,000. To make it clearer to display, we zoom in on a helicopter target.

As shown in Figure 2, the size of the circumferential image obtained by the IRCSS is much larger than that of the single-frame image obtained by the traditional staring infrared detector or the visible camera, up to 768×40,000. Directly handling the single-frame image, the efficiency of the algorithm can be slow because of the massive amount of data. In order to solve the contradiction between data quantity and algorithm efficiency, the method of sub-frame images is proposed.

The single-frame circumferential image is divided into several blocks to reduce the amount of data processed by the algorithm, and the target recognition is carried out on each image block, called a sub-frame image. According to the sequence of obtaining the single-frame and sub-frame image, the methods can be divided into direct and indirect acquisition. The direct acquisition means that in the imaging process, the sub-frame image is directly obtained through the rotation of the IRCSS with an equal angle, so the complete single-frame image is no longer stored. The indirect acquisition means that a single-frame is firstly obtained, and the sub-frame is then obtained through segmentation.

**Figure 3:**
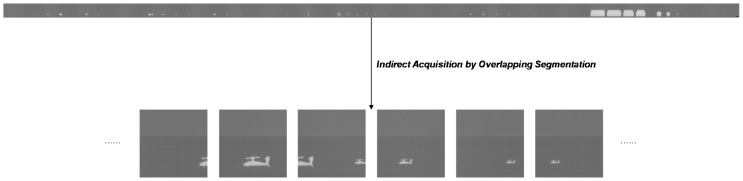
The indirect acquisition of sub-frame images by overlapping segmentation. One target is complete in at least one sub-frame image.

The existing data are complete circumferential images, so sub-frame images are obtained by indirect acquisition. To be specific, we divide a circumferential image into several 768×768 image blocks. Figure 3 shows the acquisition of subframe images. In the case of direct segmentation, some targets can be segmented into different blocks, causing problems in recognition. In order to solve this, there is an overlapping area between contiguous blocks during segmentation, such that one target is complete in at least one block. The size of the overlapping area is selected according to the maximum size of the target.

### 3.2. Infrared Target Recognition Dataset

As a type of data-driven algorithms, the DCNN needs a large amount of training data to ensure generalization performance, so that it can cope with the changes of the target itself and the scene. In the field of object recognition, training data refer to the images that contain targets and the annotations that describe the location and category of each target. It is usually expected to collect the data of different states of the targets in as many scenes as possible. However, the commonly used target recognition datasets are composed of visible (RGB) images, such as ImageNet, COCO, and VOC. In the infrared field, most of the datasets are used for target tracking, such as the VOT-TIR challenge [26,48], which consists of small sequences of infrared images containing targets. In these sequences, the target is of a single type, like pedestrians or vehicles, and the size and brightness of the target is almost unchanged, while the scene is also almost unchanged. If the training set and test set are determined by dividing a sequence randomly, the diversity between them can be too limited to guarantee the generalization of the network, although the performance on the test dataset can be noteworthy. Meanwhile, as shown in Figure 3, the background of the target in the existing data is too simple; if training is based only on this, the adaptability to the scene of the algorithm will be weak. In order to ensure that targets can be detected in infrared images of different scenes, we built an infrared target recognition dataset, including aspect orientation, size, contrast, and scene changes.

**Figure 4:**
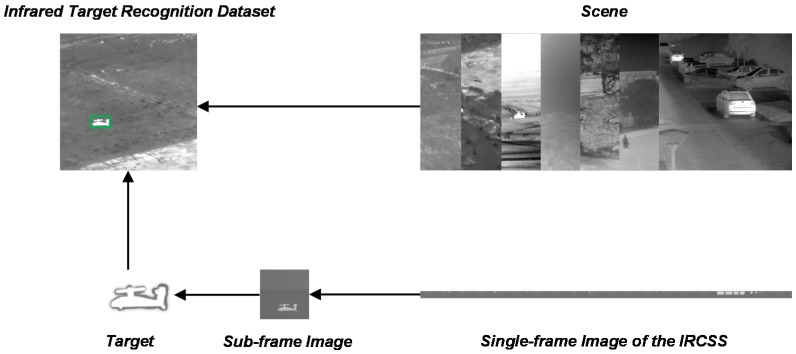
The process of building the infrared target recognition dataset. After getting the sub-frame image, we separated the target. The target was then embedded into the scene images.

As shown in Figure 4, we separated targets from the existing infrared data, including two-aspect orientations and 12 contrasts of each types of targets, and selected frames as the background from image sequences of seven types of scenes [26,49], including a road, trees, a desert, grassland, a mountain, buildings, and cars, so that there was still diversity among backgrounds of the same scene.

**Figure 5:**
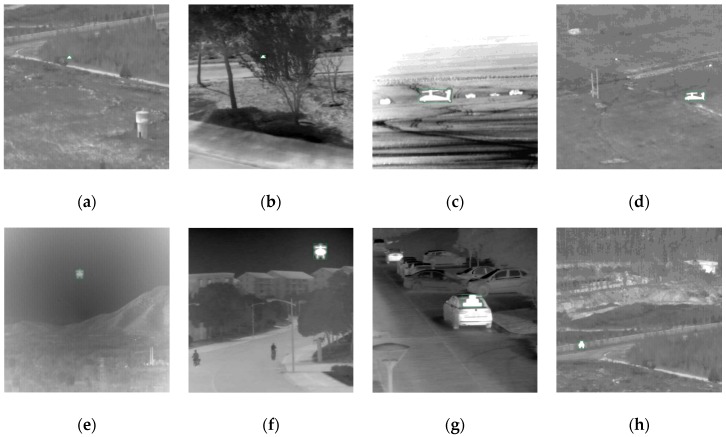
Some of the infrared target recognition dataset, including changes of target type, aspect orientation (front and side), size, contrast, and scene (road (**a**), (**h**), tree (**b**), desert (**c**), grassland (**d**), mountain (**e**), building (**f**), and car (**g**)).

When embedded in the background, each target was scaled to four sizes to simulate different distances from the detector. Thus, each type of scene contains 192 images, and the dataset has a total of 1344 images, some of which are shown in Figure 5. Compared with ImageNet and COCO, the dataset we built is too small, so we performed data augmentation, and details are shown in Section 4.

If we train on our dataset from scratch, it will easily lead to overfitting. The results in Section 4 verify this assumption. Therefore, the cross-domain transfer learning is utilized. The network used for target recognition includes a backbone network for obtaining feature maps of the input image. After training, the features extracted by the shallow layer of the backbone are common for different targets, which generally are structural features, such as edges and angles [50]. Therefore, the weight obtained from training on a large dataset, called the source domain, can be transferred to the backbone network, and we continue training with our customized dataset, called the target domain, to finetune the weight. In this way, we can not only enhance the generalization ability of the DCNN-based algorithm in the target domain but also avoid overfitting. 

Different source domain can produce different initial weights, and their finetuning effect on the target domain can also be different. In [51], the distribution of the relative size of the target in the ImageNet and COCO dataset was statistically analyzed. In ImageNet, the median relative size of the target is 0.556, while it is 0.106 in COCO, which means there are more small targets in the COCO dataset. Additionally, in COCO, the relative sizes of the maximum 10% target and the minimum 10% target differ by 20 times, which is much more than that of ImageNet, which means targets in the COCO dataset have a more extreme scale variation. In Section 4, we exploited the optimal cross-domain transfer learning strategy by experiments.

### 3.3. Smoother L1

When training a DCNN for target recognition, we define a multi-task loss function to solve both classification and localization:

(1) $$L=L_{cls}+L_{loc}.$$

The target location is realized by bounding box regression, and the objective function is a distance function between the prediction and the ground truth, which is also the target of regression, of the network:

(2) $$L_{loc}=\underset{i}{\sum }\underset{D\in \left\{x,y,w,h\right\}}{\sum }distance\left(p_{D}^{i},\;t_{D}^{i}\right).$$

Here, i represents the index of a region proposal participating in the regression, and we drop the superscript unless it is needed; D represents the four dimensions of a box coordinate, which is the abscissa and ordinate of the center of box and the width and height of the box; p represents the prediction; t represents the target. The specific definitions are as follows:

(3) $$\{\begin{array}{c} p_{x}=\omega_{x}^{T}\phi \left(r\right)\\ p_{y}=\omega_{y}^{T}\phi \left(r\right)\\ p_{w}=\omega_{w}^{T}\phi \left(r\right)\\ p_{h}=\omega_{h}^{T}\phi \left(r\right) \end{array}$$

(4) $$\{\begin{array}{l} t_{x}=\frac{G_{x}-R_{x}}{R_{w}}\\ t_{y}=\frac{G_{y}-R_{y}}{R_{h}}\\ t_{w}=\log\left(\frac{G_{w}}{R_{w}}\right)\\ t_{h}=\log\left(\frac{G_{h}}{R_{h}}\right) \end{array}$$

where $\omega_{D}$ (where D is one of x, y, w, h) represents the network parameters to be learned; ϕ(·) represents the calculation of the DCNN, r represents the region proposal, and ϕ(r) represents the features calculated by the DCNN of a region proposal; $R_{D}$ represents the coordinate of a region proposal; $G_{D}$ represents the coordinate of the corresponding ground-truth box.

In training, the gradient descent is utilized to minimize the distance between prediction and target, which can also be called loss. We take 1 as the boundary of error. Hence, each sample can be classified as an inlier (<1) or outlier (>1). For the definition of loss functions, the L2 loss is adopted in the RCNN [52].

(5) $$L2\;\mathrm{Loss}=x^{2}.$$

Because of the unlimited gradient of the L2 norm, the learning rate needs to be set very carefully in training to avoid the gradient explosion caused by outliers. In order to enhance the robustness of the loss function, smooth L1 is first utilized in Fast RCNN [53], which connects L2 with L1 by taking 1 as the boundary.

(6) L1 Loss=|x| 

(7) $$\mathrm{smooth}\;L1=\{\begin{array}{c} 0.5x^{2},\left|x\right|<1\\ \left|x\right|-0.5,others \end{array}.$$

Thus, both the undifferentiability of L1 at 0 and the sensitivity of L2 to outliers are solved. However, during training, we realized that the value of gradient participates in the update of network parameters rather than the value of loss function. Consequently, we should pay more attention to the gradient when designing the loss function. In this paper, we propose a smoother L1.

**Figure 6:**
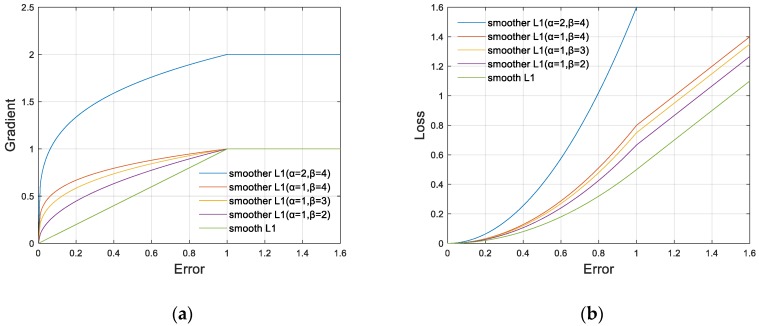
The curves of the smoother L1 with different α and β. We set α to control the gradient of outliers, and β to control the changing trend of the gradient of inliers. (**a**) The gradient curve of the smoother L1. (**b**) The loss curve of the smoother L1.

As shown in Figure 6, compared with the smooth L1, the gradient of the smoother L1 changes from a linear function to a power function for inliers and remains as a constant for outliers. By this design, the nonlinearity, which can be regarded as the core of deep learning, of the network is enhanced. On the other hand, the transition of the gradient between outliers and inliers becomes smoother to avoid the large change of the gradient during the training, which is generally considered harmful to training. 

Meanwhile, according to the ablation studies, smoother L1 inherently alleviates the imbalance between classification loss and localization loss. The gradient formula is as follow:

(8) $$\frac{\partial \mathrm{smoother}\;L1}{\partial x}=\{\begin{array}{c} \alpha {\left|x\right|}^{1/\beta },\left|x\right|<1\\ \alpha ,others \end{array},$$

where α controls the gradient of outliers, and β controls the changing trend of the gradient of inliers. The larger or smaller the β, the closer it is to L1 loss or the smooth L1. L1 loss can be regarded as the smoother L1 as α=1, β→∞, and the smooth L1 can be regarded as the smoother L1 as α=1, β=1. For the optimal setting of α and β, we did a coarse grid search in the experiment. We integrated the gradient formula to obtain the formula of the smoother L1:

(9) $$\mathrm{smoother}\;L1=\{\begin{array}{c} \frac{\alpha \cdot \beta }{\beta +1}{\left|x\right|}^{\frac{\beta +1}{\beta }},\left|x\right|<1\\ \alpha \cdot \left|x\right|-\frac{\alpha }{\beta +1},others \end{array}.$$

## 4. Experiments

### 4.1. Implementation Details

**Figure 7:**
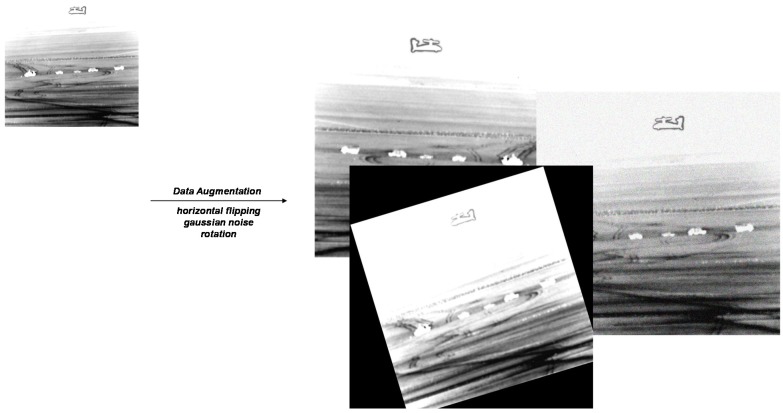
The data augmentation for each image, including horizontal flipping, Gaussian noise, rotation.

We adopt data augmentation to expand the quantity of images. As shown in Figure 7, we perform horizontal flipping, Gaussian noise, rotation for each image. The dataset eventually contains 4032 images. It is divided into 2822 images for training, 403 images for validation, and 807 images for testing. All experiments are implemented on a Lenovo Linux PC with an Nvidia RTX2060 GPU and Intel i7-9750 CPU. If not specifically noted, the batch size is set to 2 and every epoch contains 1411 iterations. We train all networks for 12 epochs, with the learning rate increasing linearly to 0.0025 in the first 500 iterations and decreasing by 0.1 after 8 and 11 epochs, respectively.

The evaluation metrics of recognition are standard COCO-style average precision (AP) [13], which is a mixture metric of widely used precision and recall. Specifically, as defined in Table 1, for the predicted bounding boxes of the same category, selecting an Intersection-over-Union (IoU) threshold $T_{\alpha }$ and then setting the confidence of each box to another threshold $T_{\beta }$, we classify each box as true positive (TP), false positive (FP), true negative (TN), and false negative (FN). Some ground truth may not have the corresponding prediction boxes; if only the prediction is judged, some FN may be missed. Therefore, following the formulas below, we calculate all the precision and recall metrics to draw the precision-recall curve. AP is the area under the curve. At last, we calculate the average value of all the AP values for all the classes in the dataset, denoted as AP_α_. In the paper, we choose mAP (the average AP over 10 IoU thresholds 0.5:0.05:0.95), which is the primary metric, AP_50_ (the AP on IoU threshold 0.5), and AP_75_ (the AP on IoU threshold 0.75). The higher the AP, the better the performance.

(10) $$precision=\frac{TP}{TP+FP}$$

(11) $$recall=\frac{TP}{TP+FN}=\frac{TP}{GT}.$$

With respect to the network, if not specifically noted, the backbone part is ResNet50 [37] that is introduced in Table 2, the feature fusion part is FPN [15], the region proposal network and the RCNN subnet follows the design of the Faster RCNN [6], and the region proposals are processed by RoI align [17]. All the hyper-parameters follow the settings of the Faster RCNN [16].

### 4.2. Comparison of Methods

We performed a comparison with SSD [41], RetinaNet [55], Faster RCNN [16], and Faster RCNN+FPN [15] to evaluate the recognition performance of our proposed method in Table 3.

Our method achieves 82.7 mAP on the test dataset. Compared with the one-stage methods (SSD [41] and RetinaNet [55]), our two-stage recognition method obtained a significant improvement. Compared with the Faster RCNN [16], we achieved a 3.0-point-higher mAP. When adding the FPN [15], we still improved the mAP by 1.2 points, which was thanks to the new loss function—the smoother L1. From the improvement of AP_50_ and AP_75_, we knew that our method could achieve target localization with higher precision. 

### 4.3. Exploiting the Optimal Cross-Domain Transfer Learning Strategy

#### 4.3.1. Weight Initialization

In the experiments, we compared the recognition performance of different weight initialization, including Xavier [56], the pre-trained weight on COCO [13] and ImageNet [12] dataset. The result is shown in Table 4.

**Figure 8:**
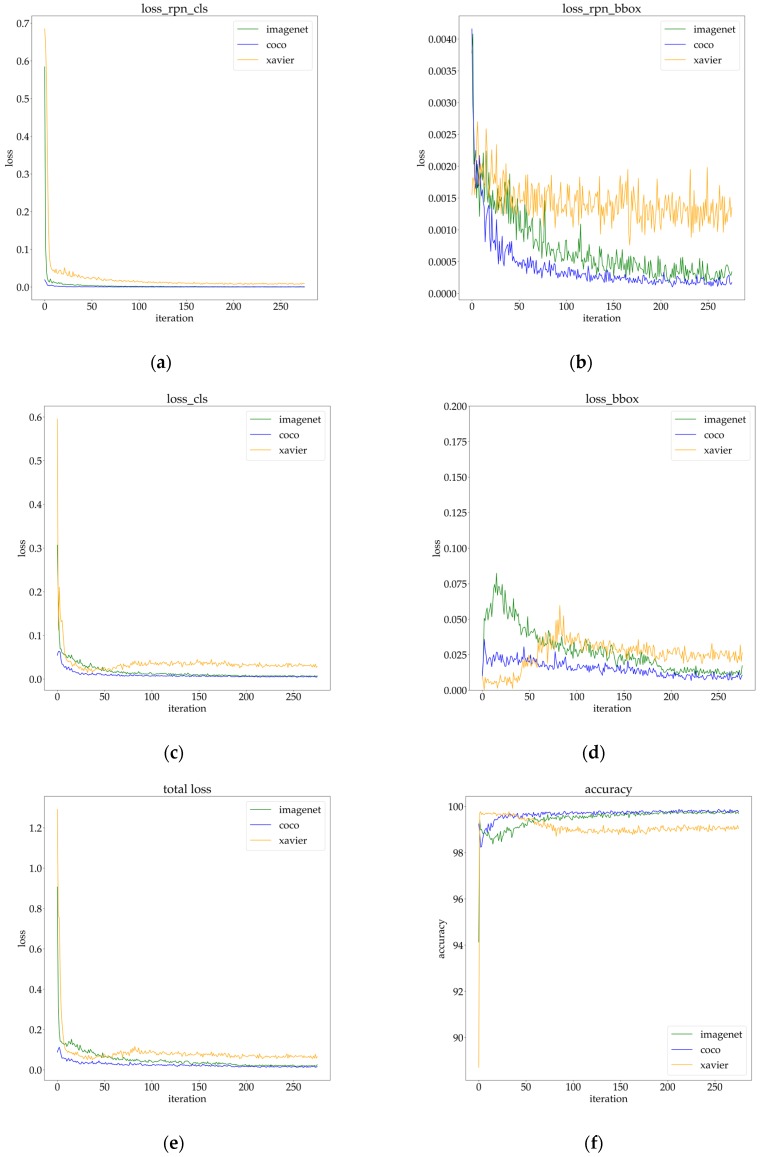
The visualization of training: (**a**) classification loss in RPN; (**b**) localization loss in RPN; (**c**) classification loss; (**d**) localization loss in bounding box regression; (**e**) total loss; (**f**) accuracy.

When the network was initialized by Xavier, the trained detector failed to detect any targets on the validation dataset under the same training setting with others. As shown in Figure 8, regardless of the method of weight initialization, the classification loss could be reduced to quite a low level.

However, the Xavier initialization could not bring about a decrease in localization loss. Meanwhile, because classification loss was much larger than localization loss, the overall loss still showed a downward trend. We conjectured that the too-small size of the training dataset led to serious overfitting. On the other hand, even though there were significant differences between source domain datasets (COCO and ImageNet) and our customized dataset, transfer learning still worked well. We observed that the pre-trained weight of COCO brought down the loss to a lower level and had a better recognition performance in all metrics than that of ImageNet. We thought that the COCO pre-trained weight was more suitable for recognition network initialization because of more small targets and a broader range of target sizes in the COCO dataset, as mentioned in Section 3.2.

#### 4.3.2. Frozen Stages

We compared the effect of different frozen stages in the network on time consumption and recognition performance. Table 5 shows the result.

We observed that the training time decreased as the number of frozen stages increased, and the recognition results also decreased. Once a stage was frozen, it would no longer participate in the update of network parameters, thus saving the time. At the same time, the network could not have a better adaptation to the input image and thus could not extract more discriminative features. There was an 18-minute difference in time consumption if the first stage was frozen (as opposed to no freezing), but the recognition results were similar. 

Therefore, in the other experiments, the network was initialized by the COCO pre-trained weight, of which the first stage was frozen during the training. 

### 4.4. Ablation Studies on the Smoother L1

For the best setting in the smoother L1, the ablation studies are shown in Table 6.

**Figure 9:**
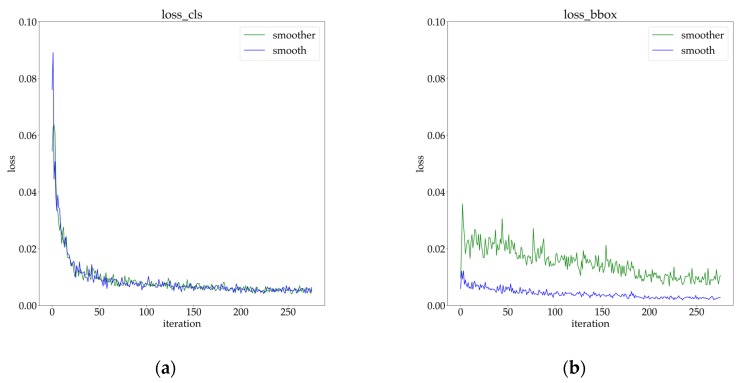
The visualization of training with the smoother L1 (α=2, β=2) and the smooth L1: (**a**) classification loss; (**b**) localization loss in bounding box regression.

We know that α controls the gradient of outliers, and β controls the changing trend of the gradient of inliers according to Section 3.3. From another perspective, the change of α could be regarded as rebalancing the classification loss and localization loss. Furthermore, the change in β could be regarded as rebalancing the localization loss of inliers and outliers. As shown in Figure 9, we observed that the smoother L1 caused the localization loss to increase, which alleviated the imbalance between classification loss and localization loss. Benefiting from a more symmetrical multi-task loss function, the network equipped with the smoother L1 could bring a 1.0-point-higher AP than the smooth L1 baseline.

### 4.5. Scene Adaptability of the DCNN-Based Method

Target recognition tasks are faced with various scenes, but the dataset cannot contain all types. In order to observe the adaptability to different infrared scenes of our DCNN-based method, and to explore whether the trained network learns the scene information or target information that we prefer, the following experiment was conducted. Six types of scenes were taken for training, and the remaining type was taken as the test dataset. A total of seven experiments were conducted.

**Figure 10:**
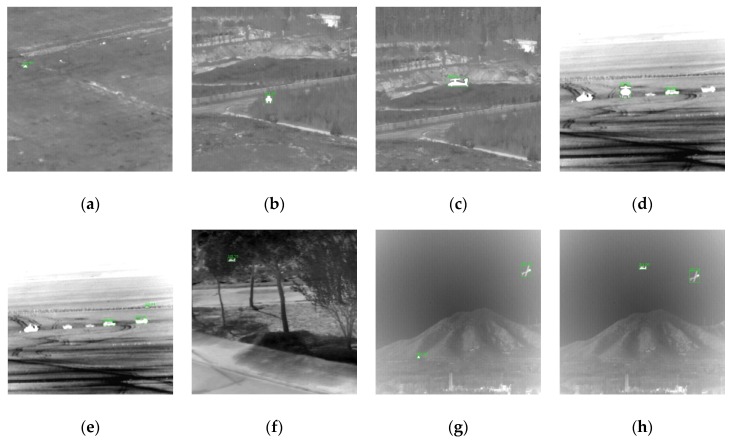
Some of the recognition results. The target is in the grassland (**a**), road (**b**), (**c**), desert (**d**), (**e**), trees (**f**), and mountains (**g**), (**h**). In (**d**), (**e**), (**g**), and (**h**), the original armored vehicles and aircraft in the scene were also recognized.

As shown in Table 7, when processing the scene that did not appear during the training, the network could still detect the target, but the performance of recognition decreased. Some of the recognition results are shown in Figure 10. We noticed that the network could recognize not only the specified target but also the original object in the scene image, such as the aircraft in the grassland and mountain scenes, and armored vehicles in the desert. It was apparent that the network could not correctly classify them because of the absence of their annotations, and they were considered as false alarms when calculating the metrics of recognition. We conjectured that the reason why the network could recognize these unknown targets in unknown scenes was that they had similar contours to the specified target; i.e., the network performed reorganization by information from the target itself instead of by speculating the location and category of the target based on the scene information. Because the object in the infrared image often lacks texture information, the characteristic of relying on contour information is noteworthy, and the designed network should pay more attention to learning contour information when recognizing the infrared target.

## 5. Conclusions and Prospect

In this paper, we propose a DCNN-based method to address end-to-end targets recognition in the IRCSS. The recognition accuracy reaches 82.7 mAP, proving the feasibility of the method. In order to solve the contradiction between the data quantity caused by the large size imaging results and the operation efficiency of the algorithm, direct and indirect acquisition of sub-frame images is proposed, and the indirect acquisition method with overlapping segmentation is selected according to the existing data in this paper. At the same time, we build an infrared target recognition dataset to both deal with the shortage of recognition data in the infrared field and enhance the adaptability of the algorithm in various scenes. During the training, on the one hand, the optimal cross-domain transfer learning strategy is exploited, including the analysis of the effect of ImageNet and COCO pre-trained weights on the recognition results and the optimal number of network frozen stages. On the other hand, through observation and analysis of the classification and localization loss, we design a smoother L1 loss function in bounding box regression and existing loss functions can be unified as specific values. Without significantly increasing the amount of calculation, it effectively improves recognition performance.

Some prospects for future work are given. Firstly, due to the low resolution of infrared images, we seek a special super-resolution algorithm as a pre-processing process in target recognition. Secondly, in the process of cross-domain transfer learning, we can adopt domain adaptation to further alleviate the performance degradation caused by the diversity between visible and infrared images. Thirdly, the method proposed in this paper has the background of practical demand, so we will continue to study how the method can be deployed on the embedded hardware platform to realize real-time automatic recognition of targets with high effectiveness both on accuracy and efficiency. 

## Figures and Tables

**Table 1:** The definition of true positive (TP), false positive (FP), true negative (TN), and false negative (FN) [54].

	$\mathrm{Confidence}\;>\;T_{\beta }$	$\mathrm{Confidence}\;<\;T_{\beta }$
**IoU >** $T_{\alpha }$	TP	FN
**IoU <** $\;T_{\alpha }$ **or** Repetitive recognition ^*^	FP	TN

* Corresponding to the same ground truth; if the IoU of multiply predicted boxes is larger than the threshold, only the bounding box with the largest IoU is considered the TP; others are considered FP.

**Table 2:** The architecture of the backbone part in the network: ResNet50 [37].

Layer Name	Stage0	Stage1	Stage2	Stage3	Stage4
**Operation** ^*^	7×7, 64, 2, 3 maxpool (3×3, 2, 1)	$\left[\begin{array}{c} 1\times 1,\;64\\ 3\times 3,\;64\\ 1\times 1,\;256 \end{array}\right]\times 3$	$\left[\begin{array}{c} 1\times 1,\;128\\ 3\times 3,\;128\\ 1\times 1,\;512 \end{array}\right]\times 4$	$\left[\begin{array}{c} 1\times 1,\;256\\ 3\times 3,\;256\\ 1\times 1,\;1024 \end{array}\right]\times 6$	$\left[\begin{array}{c} 1\times 1,\;512\\ 3\times 3,\;512\\ 1\times 1,\;2048 \end{array}\right]\times 3$

* 7×7, 64, 2, 3 means convolution kernel = 7×7, num = 64, stride = 2, and padding = 3. For all the 1×1, stride = 1, padding = 0. For the first 3×3 in Stage 2, 3, 4, stride = 2, padding = 1; for other 3×3, stride = 1, padding = 1. Every convolution is followed by batch normalization and ReLU, except the last one in every stage is only followed by batch normalization. In the block, there is a shortcut that directly connects the input with the output, and batch normalization follows the addition.

**Table 3:** Comparison of the different methods on the test dataset.

Method	Test		
	mAP	AP_50_	AP_75_
SSD(VGG)	72.5	90.5	85.8
RetinaNet	78.3	97.2	90.5
Faster RCNN	79.7	97.9	91.6
Faster RCNN+FPN	81.5	98.0	93.7
Ours	**82.7**	98.1	95.2

**Table 4:** Comparison of different methods of weight initialization on the validation dataset.

Weight Initialization	Validation		
	mAP	AP_50_	AP_75_
Xavier	\	\	\
ImageNet	80.1	95.9	94.0
COCO	83.7	97.0	97.0

**Table 5:** Comparison of different frozen stages in the network on the validation dataset.

Frozen Stages	Time Consumption	Validation		
		mAP	AP_50_	AP_75_
None	1 h 55 min	83.6	97.5	96.3
1	1 h 37 min	83.7	97.0	97.0
1, 2	1 h 30 min	82.2	97.0	95.9
1, 2, 3	1 h 18 min	80.7	96.5	95.2
1, 2, 3, 4	1 h 11 min	80.1	96.5	95.4

**Table 6:** Ablation studies of the smoother L1 on the validation dataset.

Smooth L1		82.7				
	α	1	1.5	2	2.5	3
β						
**2**		83.2	83.2	83.7	83.6	82.9
**3**		82.4	83.4	83.4	83.4	83.4
**4**		82.9	83.2	83.1	82.8	82.9

**Table 7:** Recognition results of the deep convolutional neural network (DCNN)-based method on different test scenes.

Test Scene	mAP	AP_50_	AP_75_
Grassland	67.2	97.3	77.8
Mountain	79.5	97.6	95.4
Road	80.9	96.8	92.6
Trees	74.5	93.6	89.8
Desert	76.0	93.5	92.4
Buildings	78.3	92.1	91.3
Cars	76.5	94.3	92.7
